# Mechanical properties and fluid permeability of gyroid and diamond lattice structures for intervertebral devices: functional requirements and comparative analysis

**DOI:** 10.1080/14686996.2021.1907222

**Published:** 2021-04-21

**Authors:** Anatolie Timercan, Vadim Sheremetyev, Vladimir Brailovski

**Affiliations:** a Department of Mechanical Engineering, École de Technologie Supérieure, Montreal, Quebec, Canada; b Metal Forming Department, National University of Science and Technology MISiS, Moscow, Russia

**Keywords:** Lattice structures, additive manufacturing, geometric analysis, mechanical testing, fluid permeability, 10 Engineering and Structural material, 102 Porous / Nanoporous / Nanostructured materials, 106 Metallic materials, 211 Scaffold / Tissue engineering / Drug delivery, 305 Plasma / Laser processing

## Abstract

Current intervertebral fusion devices present multiple complication risks such as a lack of fixation, device migration and subsidence. An emerging solution to these problems is the use of additively manufactured lattice structures that are mechanically compliant and permeable to fluids, thus promoting osseointegration and reducing complication risks. Strut-based diamond and sheet-based gyroid lattice configurations having a pore diameter of 750 µm and levels of porosity of 60, 70 and 80% are designed and manufactured from Ti-6Al-4V alloy using laser powder bed fusion. The resulting structures are CT–scanned, compression tested and subjected to fluid permeability evaluation. The stiffness of both structures (1.9–4.8 GPa) is comparable to that of bone, while their mechanical resistance (52–160 MPa) is greater than that of vertebrae (3–6 MPa), thus decreasing the risks of wither bone or implant failure. The fluid permeability (5–57 × 10^−9^ m^2^) and surface-to-volume ratios (~3) of both lattice structures are close to those of vertebrae. This study shows that both types of lattice structures can be produced to suit the application specifications within certain limits imposed by physical and equipment-related constraints, providing potential solutions for reducing the complication rate of spinal devices by offering a better fixation through osseointegration.

## Graphical abstract

**Figure:**
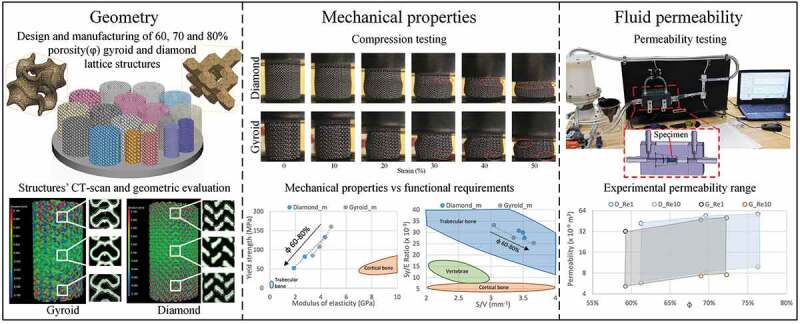

## Introduction

According to Statistics Canada, spine health problems are amongst the most common sources of chronic pain in Canadians requiring intervertebral fusion surgery in extreme cases [1]. This procedure involves the fixation of two or more adjacent vertebrae using spinal instrumentation such as spinal rods and spinal cages. A number of different cage designs are commonly used by surgeons, depending on the surgery site, the illness severity, and the preferred surgical technique. Larger cages (threaded cylindrical and box-shaped) are employed in anterior and lateral intervertebral fusion approaches, while smaller cages (bullet- and kidney-shaped) are preferred for posterior and transforaminal intervertebral fusion [2,3]. It must, however, be noted that the currently used commercial intervertebral cages present different complication risks, such as a lack of fixation, cage migration and subsidence [4,5]. The reported complication rates vary from 6 to 30%, depending on the cage type, the material, the size, and the surgical technique [6,7].

Leading efforts to reduce the above problems concentrate on the employment of functional materials, such as superelastic alloys [8], and the development of porous [9,10] and patient-specific [11,12] spinal devices. Superelastic alloys have a lower modulus of elasticity than the traditionally used metallic implant materials. The plateau–like hysteretic behavior of these alloys, being closer to the mechanical behavior of bone, may reduce the risk of stress shielding [13]. Porous structures are considered as good candidates for this application because of their lower stiffness than their bulk equivalents [14,15]. Moreover, porous structures with open interconnected pores are permeable to fluids, which could improve their union with the surrounding tissues via osseointegration [15–18]. Finally, patient-specific devices have the advantage of being based on patients’ anatomy extracted from body scans, and thus promise a better fit and function due to their personalized geometries [19]. An appropriate synergetic combination of all these attributes, spanning from the material behavior at the microscale to the structural arrangement at the mesoscale, and to the device geometry at the macroscale, could help to reach the ultimate objective of improving the clinical performances of spinal cages. This work focuses on the mesoscale aspect of spinal cages, i.e. on the design and performances of porous structures used for their manufacture.

Porous structures may be stochastic (foams) or ordered (cellular) structures. The foams used to be more common due to their production simplicity. However, with the advent of additive manufacturing, allowing the production of complex geometries, cellular structures have gained in interest and popularity [20]. Cellular structures, also known as lattice structures, are defined by the 3D repetition of a given unit cell. As compared to their stochastic counterparts, the properties of lattice structures can more readily be controlled by tuning the lattice geometry, which makes them easier to model and simulate, and allows for a greater manufacturing repeatability.

Lattice structures can be classified in three main categories in terms of their geometry: strut-, skeletal- and sheet-based geometries [21]. Strut-based structures are composed of linear rods with constant or variable cross-sections interconnected at the nodes. Among their advantages is the simplicity of creation. These lattice structures are, however, prone to stress concentrations at the nodes. Skeletal and sheet-based structures are built using triply periodic minimal surface equations based on sinusoidal functions. These lattice structures have smoother transitions, and, therefore, less pronounced stress riser effects. It is worth noting that at high porosity levels, manufacturing-induced defects can act as additional stress risers distributed over the entire structure for all the lattice categories, and their contribution can play a significant role when the strut thickness approaches the smallest feature resolution of the manufacturing system [22].

Lattices may be characterized by a combination of three sets of attributes: geometric, mechanical and fluid-related, all the attributes that influence the service performance of lattices as implant materials. Geometric attributes include the strut/sheet thickness, the cell size, the pore diameter, the surface area, the total volume and the level of porosity. These parameters are co-dependent and directly affect the mechanical properties such as the strength and stiffness, as well as the fluid permeability. For example, the apparent modulus of elasticity and yield strength of lattice structures can be calculated as functions of porosity using scaling relations proposed by Gibson and Ashby (Equations (1) and (2)):

(1) $$E_{app}=E_{b}\;*\;C_{1}{\left(1-\varphi \right)}^{n_{1}}$$

(2) $$S_{y_{app}}=S_{y,b}\;*\;C_{2}{\left(1-\varphi \right)}^{n_{2}}$$

where *E_b_* and *S_y,b_* are the modulus of elasticity and the yield strength of the bulk material, and *C* and n are the empirically determined coefficients.

Similarly, fluid permeability can be related to porosity via the Kozeny-Carman equation (Equation (3)), which is used to approximate the absolute permeability of soil in the earth sciences [23]:

(3) $$k=\frac{\varphi^{3}}{c\;*\;{\left(1-\varphi \right)}^{2}\;*\;S^{2}}$$

where *k* is the absolute permeability, *c* is the Kozeny–Carman constant, and *S* is the specific surface area of the material.

In Equations (1)–(3), the porosity *φ* is defined as the volume of voids divided by the total volume of the structure (Equation (4)):

(4) $$\varphi \left(\%\right)=\frac{V_{void}}{V_{total}}*100=\left(1-\frac{V_{material}}{V_{total}}\right)*100$$

In order to evaluate the performances of various lattice configurations and allow choosing the most appropriate one for a selected application (spinal cages, in our case), it is necessary to define the functional requirements and the range of acceptable properties for this application.

## Functional requirements to intervertebral cages

The human spine is complex and does not accommodate a one-size-fits–all approach. It can be divided into three main regions, namely, the cervical, thoracic and lumbar zones, which progressively support a greater load, and are therefore increasingly larger [24]. Morphological analyses of the vertebrae at all levels have been carried out, and their average dimensions identified, as shown in Table 1. One of the limitations of the related studies is that they are based on an analysis of spines of deceased persons, and consequently, are biased toward older population [25–27].

**Table 1.** Average dimensions of vertebrae at the cervical, thoracic and lumbar levels [25–27]

	Transverse diameter [mm]	Sagittal diameter [mm]	Endplate rim thickness [mm]	Vertebral height [mm]
Cervical	12–29	12–24	1–6	10–30
Thoracic	12–44	12–39	1–9	12–45
Lumbar	43–50	29–35	2–12	26–27

The load supported by the vertebrae and the intervertebral discs varies, depending on the activity performed and the body position. The load acts as a combination of compression, bending and torsion. The resistance of the vertebrae and disks is most easily tested in compression. It was found that the vertebral compression failure load is in the 2–6 kN range, or around 3–6 MPa, when distributed over the cross–section [24]. For comparison, trabecular bone has a yield strength of 0.2–10.5 MPa [28], while that of cortical bone, of 42–176 MPa [29,30]. The modulus of elasticity of the vertebrae was measured to be 0.374 GPa [31], which is situated much closer to the upper limit value of the apparent modulus of trabecular bone (0.043–0.165 GPa) [32] than to that of cortical bone (7–30 GPa) [30]. Both mechanical attributes of human vertebrae, resistance and stiffness, are therefore defined mainly by those of trabecular bones, which is explained by a relatively small thickness of cortical bone in these structures (~300 µm) [33].

From these values, the strength-to–stiffness ratio (*Sy*/*E*) for the vertebrae ranges from 8 to 16 × 10^–3^; for trabecular bone, it ranges from 5 to 60 × 10^−3^, and for cortical bone, it is ~6 × 10^−3^. It can be stated that the greater the *Sy*/*E* ratio of engineered lattice structures, the better they are suited for the application, providing the resistance of these structures exceeds that of surrounding tissues. Maximizing this ratio, while preventing mechanical failure, would allow maintaining the stiffness of a lattice structure as close as possible to that of the vertebrae, thus reducing the stress shielding effect which occurs when the implant stiffness is higher than that of bone in the site of implantation.

To stimulate osseointegration, lattice structures must respect certain criteria with regards to the porosity level, the pore size and permeability. In human bones, these parameters depend on their location, type of loading and bone quality [34]. For example, the trabecular bone in the vertebrae has an apparent porosity ranging from 70 to 97% [28,32], which is significantly higher than the 30–70% porosity range recommended by the FDA for porous coated knee, hip and shoulder implants [35]. Finally, to promote the ingrowth of bone instead of connective tissue, a pore size range of 100 to 1000 µm is recommended [16–18,30]. As well, to select a better candidate for bone tissue scaffolds from different lattice structures, the surface-to–volume (*S/V*) ratio can be used [16,17], since the greater this ratio, the greater the surface available to host the ingrowth tissue. This ratio in bones varies from 1 to 6 mm^−1^, and in vertebrae, it varies from 2 to 3 mm^−1^ [36–38].

Regarding the permeability of bones to fluids, which ensures an adequate nutrient supply to surrounding tissues, average values reported in the literature for vertebrae vary from 0.49 to 44.5 × 10^−9^ m^2^, depending on the sample selection and preparation [39–41]. In these sources, the absolute permeability *k* is calculated using Darcy’s law by measuring the pressure drop and fluid velocity through a bone sample (Equation (5)).

(5) $$k=\frac{Q\;*\;\mu }{A\;*\;\frac{\Delta p}{L}}$$

where *Q* is the flow rate (m^3^/s), *µ* is the dynamic viscosity (Pa·s), *A* is the bone sample cross-section (m^2^), *Δp* is the pressure drop across the sample (Pa), and *L* is the sample length (m).

**Figure 1.**
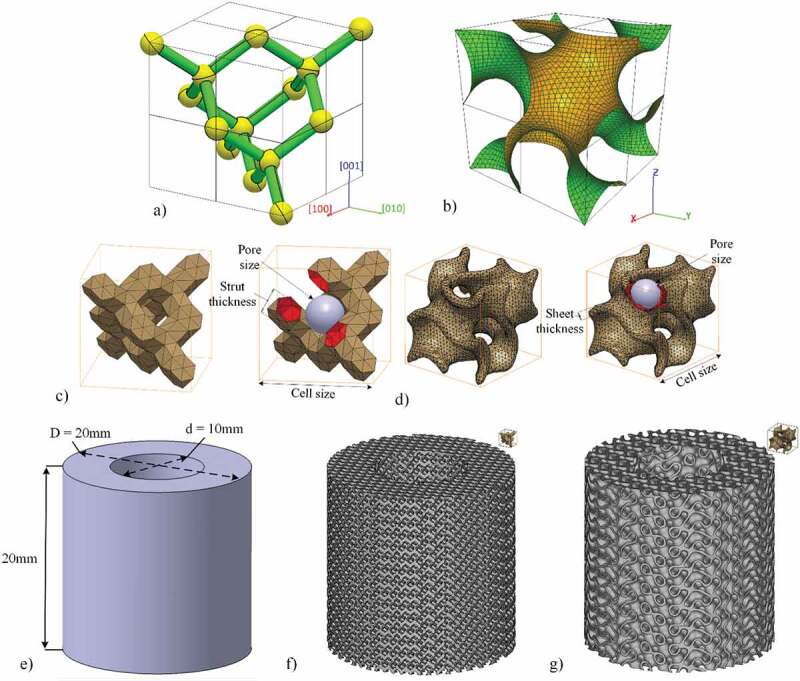
Default unit cell disposition of the (a) diamond lattice (Miller indices are added, for convenience), (b) gyroid lattice. Unit cell as generated in this study and the control parameters (c) diamond lattice and (d) gyroid lattice. Specimens for compression testing: (e) CAD, (f) diamond lattice and (g) gyroid lattice

The objective of this study is to compare two competing lattice structure geometries, namely the strut-based diamond lattice and the sheet-based gyroid lattice, for use in spinal cages Figure 1(a,b). These structures must satisfy the pre-established functional requirements for bone replacement and need to be compared in terms of their geometric, mechanical and fluid permeability attributes. The two types of cellular structures considered in this study resemble the two principal architypes of trabecular bone structure found in the skeleton, notably rod-like and plate-like structures [34]. The diamond lattice is composed of struts connected at nodes that correspond to the carbon atom placement in a diamond. One of the advantages of the diamond lattice is its strut orientation, which is convenient for additive manufacturing since it does not require supports. This structure is one of the most studied in the literature, and can be generated using a number of commercial software applications, including *Magics* and *3matic* from the *Materialise (Leuven, Belgium)* suite, *Workbench-Material Designer* by *Ansys (Pennsylvania, USA)* and *Optistruct* by *Altair Hyperworks (Michigan, USA)*. On the other hand, the sheet-based gyroid lattice is a triply periodic minimal surface structure (TPMS) based on sinusoidal functions. To create these structures, some programming software, such as *Grasshopper* from *Rhino3D (Robert McNeel & Associates, Washington, USA)* and *MATLAB* by *MathWorks*  *(Massachusetts, USA)* could be used; software allowing the custom unit cell lattice replication, such as the *Structures Module* of *Magics* or *Simpleware* by *Synopsys (California, USA)*, could also be employed. The gyroid lattice structures were shown to have a greater surface area and mechanical resistance, but lower fluid permeability, than their strut-based equivalents with similar porosities and cell sizes [21,42,43]. It is worth noting that some recently-developed software tools such as *MSLattice (Abu Dhabi, UAE)* for example, allow the creation of structures with functionally–graded porosity, resulting in lattices with progressive morphology similar to that of bone [44–49]. However, an adequate understanding of the behavior of lattice structures with constant porosity represents a necessary prerequisite for the effective practical application of these graded materials.

To the authors’ knowledge, there are multiple studies analyzing different lattice structures on the basis of their geometry attributes and either mechanical or fluidic permeability characteristics. However, there is a lack of comprehensive studies that compare different lattice structures concurrently on the basis of all three characteristic sets, especially with a specific end-use in mind and in a significantly large range of porosity variations. Among the most recent studies, the following can be mentioned: Yu et al. [50] studied all three attributes for different lattice geometries (primitive, gyroid, bcc) but with a single porosity level, Ma et al. [51] and Bobbert et al. [52] analysed the mechanical and the mass-transport properties of lattice structures with different levels of porosity but limited their studies to either gyroid [51] or sheet TPMS structures [52]. To this end, the lattice geometry and parameters are selected in conformity with the established functional constraints. Then, the selected structures are designed and manufactured using the laser powder bed fusion additive manufacturing technology. Finally, their geometric, mechanical and fluid permeability properties are assessed using computed tomography, compression testing and permeability measurement techniques.

## Materials and methods

### Lattice selection

Both types of lattice structures can be defined by three parameters: cell size, strut/sheet thickness and pore size, two of which are independent and can be adjusted to control the porosity Figure 1(c,d). In this work, diamond structures are generated with the help of a proprietary *MATLAB* algorithm [53], which uses voxelization to create a triangular mesh of struts in the form of hexagonal prisms and connection nodes in the form of truncated tetrahedrons. To create the gyroid structures, the free-access mathematical modelling software *MathMod* is used to generate a zero-thickness mesh of the gyroid, which is then scaled and offset to the desired cell size and sheet thickness in the *CATIA V5*  *(Dassault Systèmes, France)* software environment.

Among the three main diamond cell orientations: [001], [011] and [111], the [001] is selected, since it displays a more constant cross–sectional area along the vertical direction, thus reducing the stress riser effect and improving the tensile fatigue resistance of the structure [54]. Although some recent works study the impact of the gyroid orientation on its stiffness and strength [55–57], they present conflicting results, therefore the gyroid structure orientation is kept by default, and is determined by the governing equation used for its generation (Equation (6)):

(6) $$\cos\left(x\right)*\sin\left(y\right)+\cos\left(y\right)*\sin\left(z\right)+\cos\left(z\right)*\sin\left(x\right)=0$$

For this study, diamond and gyroid lattice structures with a constant pore diameter of 750 µm and three target porosity levels of 60, 70 and 80% are designed using the lattice parameter sets shown in Table 2. The pore diameter of 750 µm, which is near the upper limit of the recommended 100–1000 μm range, is selected to favor osseointegration, while keeping pore dimensions large enough from a manufacturing constraints viewpoint [18]. The porosity window of this study, 60–80%, is determined based on the geometric limitations of gyroid structures, which have inherently larger cell sizes and thinner sheets than diamond structures of the same porosity. For example, to keep a minimum of two contiguous cells along the intravertebral height of ~5 mm [58,59], a unitary gyroid cell cannot be bigger than 2.5 mm (2500 μm), which results in a porosity of 60%. At an upper level of 80%, the minimal sheet thickness of ~100 µm of the gyroid structures is close to the manufacturing limits of most laser powder bed fusion (LPBF) additive manufacturing systems [60]. The middle value of 70% is selected to provide a minimum of three data points for the generation of scaling relations. It is worth noting that the highest level of porosity in this study corresponds to 80%; higher porosity levels are achievable by increasing the cell size or by reducing the strut thickness, as shown in [61]. The porosity and the surface-to-volume ratio of both structures are calculated by extracting the total volume, the object volume and the object surface area using the mesh analysis (Table 2).

**Table 2.** Selected diamond/gyroid lattice structure geometric parameters for a pore size of 750 μm and target porosities of 60, 70 and 80%

	Strut [µm]	Cell [µm]	Porosity [%]	*S*/*V* [mm^−1^]
Diamond	455	1485	62.2	2.66
	345	1336	71.0	2.82
	240	1193	80.9	2.81
Gyroid	305	2431	61.0	2.40
	210	2191	70.2	2.74
	125	1977	80.1	3.10

### Experimental testing

The design of hollow cylinder compression testing specimens is based on the geometry of an average cervical disc, with an outer diameter of 20 mm and an inner diameter of 10 mm. Their height is set to 20 mm in order to respect the ISO13314 standard for compression testing of cellular materials [62], which requires a height-to-diameter ratio of ~1–2. The porous specimens are generated by Boolean intersection between an oversized lattice structure and the specimens’ CAD Figure 1(e–g). Similarly, full cylinder permeability testing specimens with an outer diameter of 10 mm and a height of 20 mm are also created.

**Figure 2.**
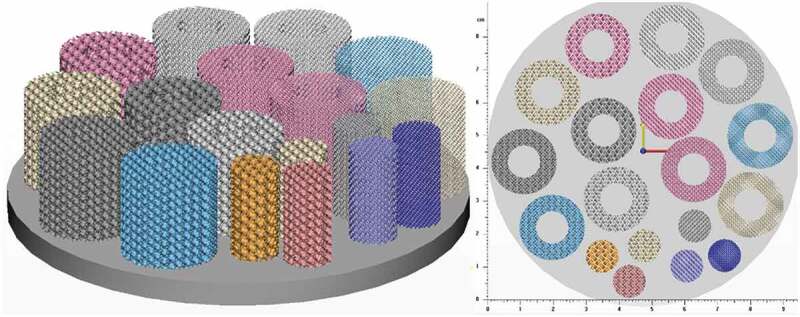
Specimen distribution on the build plate

Specimen manufacturing is carried out on a *TruPrint1000* LPBF system (*Trumpf, Ditzingen, Germany*) with a laser spot size of 30 µm. The material used in this study is Ti64–ELI which has a tabulated modulus of elasticity of 114 GPa and a yield strength of 1120 MPa [63]. The powder particle size distribution as provided by the manufacturer is 15–45 µm. The default printing parameters indicated by Trumpf are used: 155 W laser power, 1200 mm/s scanning speed, 110 µm hatching space and 20 µm layer thickness. Preliminary prints indicated a 70 µm manufacturing error, which is accounted for in the actual design of the cellular structures. Two specimens for mechanical testing and one specimen for permeability testing of the diamond and gyroid structures with target porosities of 60, 70 and 80% are manufactured, bringing a total number of specimens to 18 (Figure 2).

Prior to part separation from the build plate, a dual stage heat treatment (DSHT) under vacuum (45 min at 1010°C followed by 1 h at 850°C) is performed following the recommendations of the equipment manufacturer. Specimens are cut by wire EDM and cleaned using high-pressure water.

**Figure 3.**
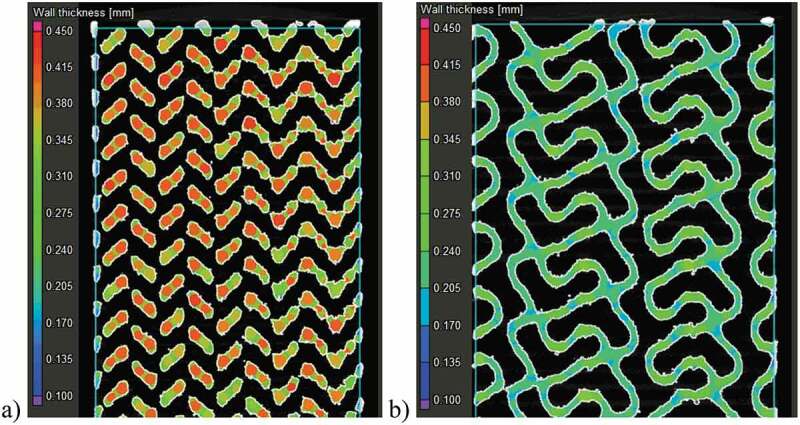
Specimen strut thickness measurements in ~70% porosity structures using the sphere method in VGStudio MAX software: (a) diamond and (b) gyroid structures

The resulting structures are scanned using an *XTH225* micro computed tomography (µ-CT) system (*Nikon, Tokyo, Japan*) with a 190 kV tube voltage, 50 µA current and 10.8 µm resolution. The scans are reconstructed using *CT Pro 3D* software (*Nikon*) to generate TIFF image stacks. *VGStudio MAX 3.1* software (*Volume Graphics, Heidelberg, Germany*) is used to convert the image stacks to volumes and compare these volumes to the respective CAD models. Analyses of the specimen geometry in terms of porosity, strut thickness and pore diameter are also carried out (Figure 3).

Next, an experimental porosity evaluation is conducted in conformity with the ASTM F2450-18 standard by calculating the volume of the matter in the total volume of the specimen measuring its mass (*Acculab  (Gottingen, Germany) L-series* scale, ± 0.0001 g) and geometry (*Mitutoyo (Kanagawa, Japan) Absolute* caliper, ± 0.01 mm) [64]. To that end, Equation (4) is used where the volume of material is calculated by dividing the specimen mass by its density (4.43 g/cm^3^ for Ti-6Al-4V), and the total volume is calculated based on the specimen diameter and height.

Two previous porosity measurements are verified using Archimedes’ technique (ASTM B963-18) [65]. To this end, specimens are weighed three times: dry, impregnated with oil (*Mobil (Texas, USA) SHC 634*), and submerged in water while oil–impregnated (*Sartorius (Gottingen, Germany) Secura 324-1s* scale, ± 0.0001 g) (Equation (7)).

(7) $$\varphi \left(\%\right)=\left(\frac{m_{oil}-m_{dry}}{(m_{oil}-m_{oil\;water})*\rho_{o}}*100\right)*\rho_{w}$$

where *m_dry_* is the mass of the dry specimen (g), *m_oil_* is the mass of the specimen impregnated with oil (g), *m_oil water_* is the mass of the oil-impregnated specimen submerged in water, *ρ_o_* is the relative density of oil (0.87) and *ρ_w_* is the water density (0.9977 g/cm^3^); both at room temperature.

Next, one specimen of each structure is placed between parallel lubricated platens and tested in compression on an *Instron  (Massachusetts, USA) 150LX* materials testing system at a displacement rate of 5 mm/min until densification onset. The compression rate falls within a recommended range of 10^−3^–10^−2^ s^−1^ [62]. The acquired force-displacement data are converted to the stress-strain diagrams using the specimen cross-section and length, and the apparent modulus of elasticity and yield strength values are then extracted.

**Figure 4.**
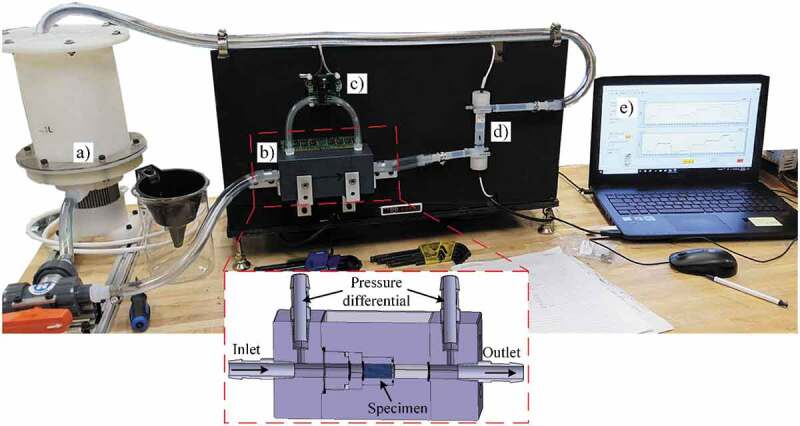
Permeability test setup: (a) mixer tank/pump assembly, (b) specimen holder, (c) differential pressure transducer, (d) flowmeter and (e) laptop with LabView for data treatment. Not shown on the image are the power supply, pump controller and data acquisition card

The structures’ permeability is measured using an in-house manufactured setup (Figure 4). Distilled water is pumped through the specimen using a *Levitronix  (Zurich, Switzerland) PTM-1* pump mixer assembly with a flow range of 0 to 17 L/min. The flow is measured using a *Leviflow  (Zurich, Switzerland) LFS-20-Z* 0 to 20 L/min sensor with an accuracy of ± 1%. Prior to testing, specimens undergo an ultrasonic bath cleaning to remove any loosely bonded particles inside the structure. To avoid bypass flow, the lateral walls of the permeability specimens are wrapped using PTFE tape. The differential pressure between the inlet and outlet ports is measured using an *Omega (Connecticut, USA) PX26* differential transducer with a capacity of ± 1 bar and an accuracy of ± 1%. An *Omega DPG4000-15–RM* high accuracy (± 0.05%) digital pressure gauge is used to calibrate the differential transducer in the 0–0.4 bar measurement range.

A *National Instruments USB–6212 (Texas, USA)* data acquisition card and the *LabView 2018 (Texas, USA)* software are used to acquire and register data from the flowmeter and the pressure transducer. Measurements are carried out from 0.4 to 2 L/min in increments of 0.1 L/min under steady state conditions, and the results obtained are approximated by a power law. Next, the flow and pressure drop values are extrapolated for the Reynolds numbers ranging from 1 to 10 to fall within the definition of the Darcy regime [52,66–68]. To calculate the Reynolds number, Equation (8) is used:

(8) $$Re=\;\frac{\rho *v_{f}*l}{\mu }$$

where *ρ* is the fluid density (997 kg/m^3^ for water), *v_f_* is the fluid velocity (m/s) also equal to flow *Q* (m^3^/s) divided by specimen cross-section *A* (m^2^), *l* is the pore diameter (m) as measured from the CT–scan and *µ* is the dynamic viscosity of the fluid (0.001 Pa·s for water).

## Results

**Figure 5.**
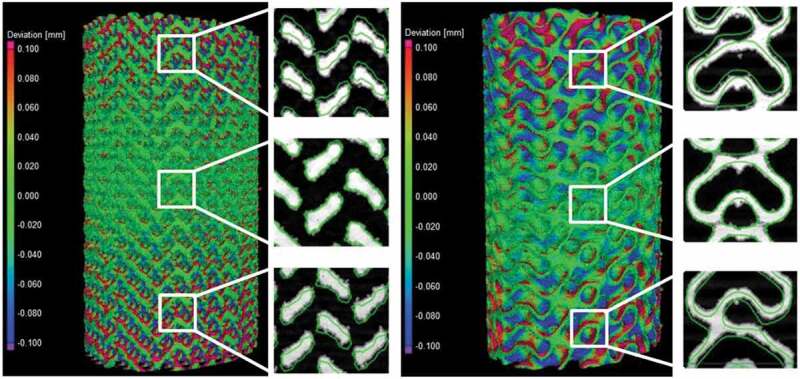
Deviation maps between the designed and manufactured 70% diamond and gyroid structures following a best-fit: the detailed views show the design in a green outline and the scanned structure in solid white

A visual comparison between the designed and manufactured geometries indicates a shrinkage in the build direction (Figure 5). All manufactured structures show deviations situated between ± 100 and ± 250 μm for 10–25% of their total surface; the remaining 75–90% of their surface falls within the ± 100 μm range of deviations from the CAD geometry. No powder plugging can be observed inside the structures which would affect the mechanical or permeability measurements.

**Figure 6.**
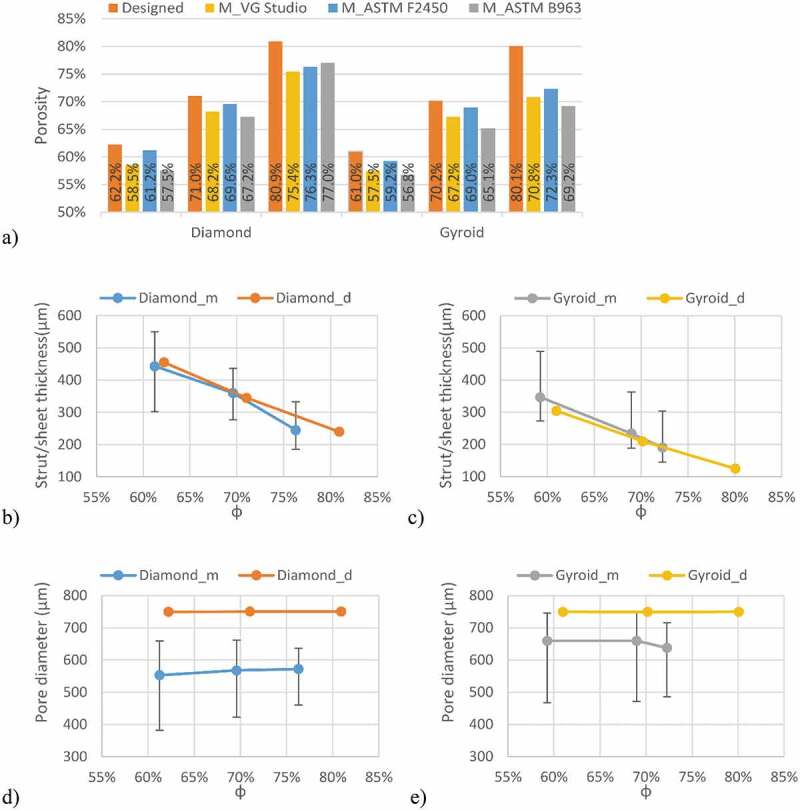
Comparison of the designed (d) and manufactured (m) diamond and gyroid structures: (a) porosity, (b) diamond strut thickness, (c) gyroid sheet thickness, (d) diamond pore diameter, and (e) gyroid pore diameter

Figure 6 summarizes the results of the geometric comparison between the designed and manufactured structures. All the manufactured structures have a slightly lower porosity than the designed ones, no matter the measurement technique (Figure 6(a)). The manufactured 60 and 70% porosity specimens are generally closer to the designed ones than the 80% specimens. The highest discrepancy among the three competing porosity measurement techniques (VG Studio, ASTM F2450 and Archimedes’ ASTM B963) corresponds to 3.9% of porosity, and the results obtained using the ASTM F2450 approach are generally higher. These last porosity values will be used for all the following representations and comparisons.

Notwithstanding with the above, the strut thicknesses of the manufactured diamond structures are closer to the designed values than those of their gyroid equivalents Figure 6(b,c). For all the structures, the pore diameters of the manufactured specimens are smaller than those of the designed ones, with the pores of the diamond structures being smaller than those of the gyroid structures Figure 6(d,e). The 80% porosity gyroid structures seem to approach the limits of the manufacturing system, since their sheet thickness and pore diameter diverge the most from their designed equivalents.

**Figure 7.**
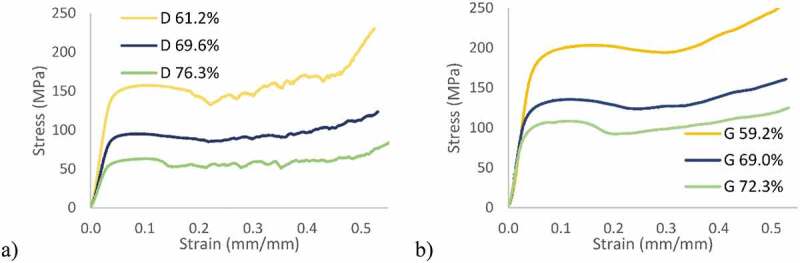
Stress-strain diagrams: (a) diamond and (b) gyroid structures

Considering the mechanical behavior Figure 7(a,b), the diamond lattice structures (Figure 7(a)) exhibit a more unstable behavior after reaching the peak stress than do their gyroid equivalents (Figure 7(b)). That indicates a sudden collapse of certain struts in the diamond structures, as opposed to the gyroid lattices, in which the cell collapse is more gradual. A similar behavior was observed by Al-Ketan, Rowshan [21] and Zhou, Zhao [48], where strut- and skeletal-based structures experienced larger stress variations following the first maximum compressive strength than the sheet-based structures.

**Figure 8.**
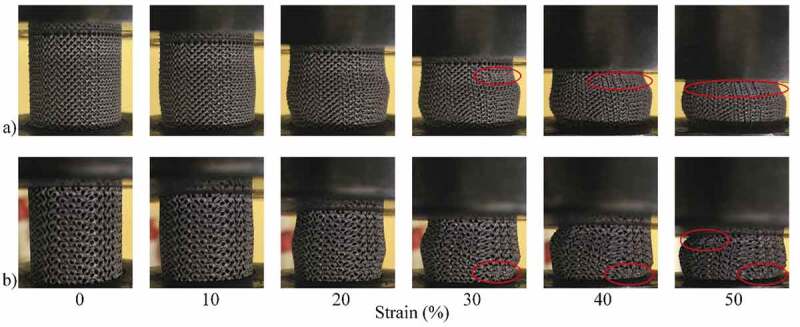
Compression testing of ~70% porosity structures: (a) diamond and (b) gyroid sample shape evolution during compression at various compression strain values. Areas where densification is observed are circled in red

**Figure 9.**
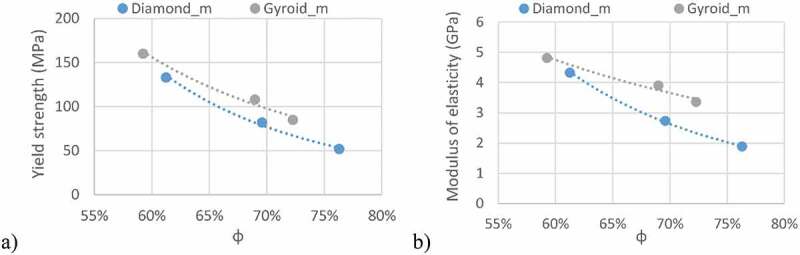
Apparent mechanical characteristics of the manufactured structures: (a) yield strength and (b) modulus of elasticity

Figure 8 illustrates the deformation of the specimens during compression testing and the onset of densification in the structures, which occurs at a strain level of ~30%. From a mechanical properties’ standpoint, it can be seen that the gyroid lattice is stiffer and stronger than its diamond counterpart for the same levels of porosity Figure 9(a,b).

**Figure 10.**
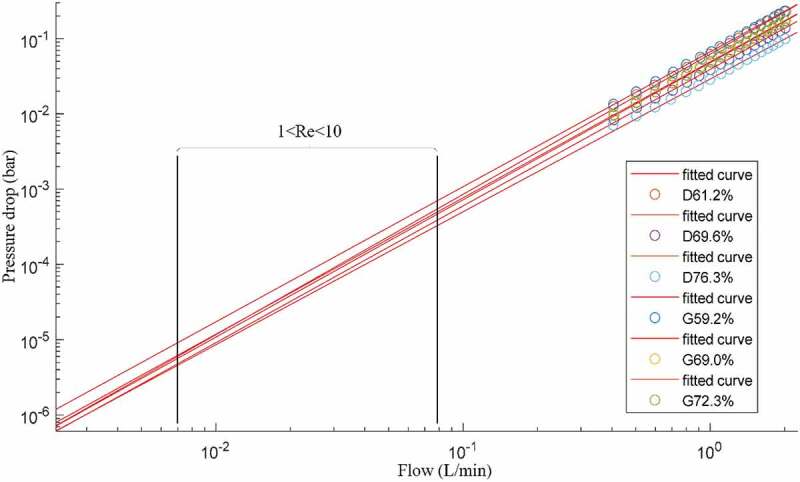
Flow and pressure drop experimental measurements, where D signifies diamond and G gyroid structures of different porosities; black rectangle delimits the extrapolated range where Reynolds number varies between 1 and 10

**Figure 11.**
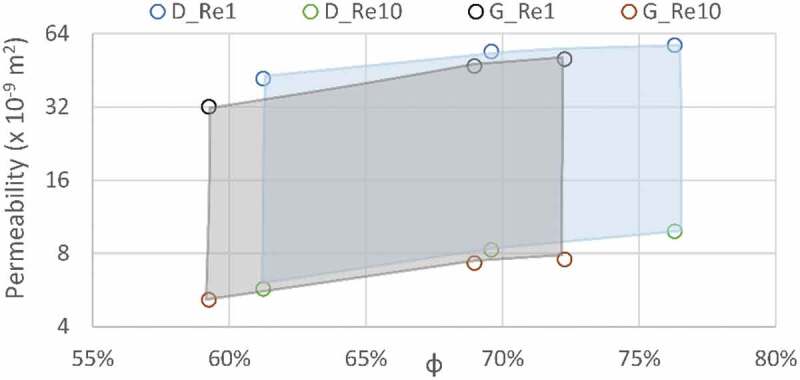
Permeability results from the extrapolated data are shown as the regions of the Darcy regime (Re1 and Re10 correspond to Reynolds numbers of 1 and 10); diamond (D) structures are presented in blue and gyroid (G) structures in grey

Figure 10 shows the experimental flow and pressure drop measurements for all the studied lattice structures along with their respective fitted power law curves. All fitted equations had R^2^ correlation factors over 99.97%. The Darcy regime corresponds to flow rates situated between 0.007 and 0.08 L/min, where the Reynolds number ranges from 1 to 10. These flow rate values are in the same range as those used for permeability testing of bone or engineered lattice structures in [40,41,52,69], and the fluid velocities (flow *Q*/area *A*) are in the same range as those used for fluid flow stimulation of bone cells [70]. The permeabilities of the manufactured structures corresponding to the Darcy regime region are plotted in Figure 11.

From the data obtained, the following scaling relations can be extracted for the yield strength, the modulus of elasticity and the permeability of both structures. These relations can help to predict the behavior of lattice structures having a constant pore size of 750 μm for varying levels of porosity. The modulus of elasticity and yield strength equations follow the Gibson and Ashby $X=C\;*\;{\left(1-\varphi \right)}^{n}$ scaling relation format (Table 3). The permeability equation fitting the Kozeny-Carman formulation yields negative R^2^ values, meaning that a horizontal line is a better fit than the formulation, and therefore, the classical Gibson and Ashby formulation is used in this case also.

**Table 3.** Scaling relations for modulus of elasticity, yield strength and permeability of the diamond and gyroid structures; Re1 and Re10 correspond to Reynolds numbers of 1 and 10

Diamond	$E_{app}=E*0.1977*{\left(1-\varphi \right)}^{1.747}$	R^2^.9965
	${S_{y}}_{app}=S_{y}*0.7427*{\left(1-\varphi \right)}^{1.937}$	R^2^.9995
	Extrapolated $k_{Re1}=23.56*10^{-9}*{\left(1-\varphi \right)}^{-0.5978}$ Extrapolated $k_{Re10}=2.159*10^{-9}*{\left(1-\varphi \right)}^{-1.031}$	R^2^.8671 R^2^.9543
Gyroid	$E_{app}=E*0.0938*{\left(1-\varphi \right)}^{0.884}$	R^2^.9826
	${S_{y}}_{app}=S_{y}*0.5865*{\left(1-\varphi \right)}^{1.569}$	R^2^.9936
	Extrapolated $k_{Re1}=10.94*10^{-9}*{\left(1-\varphi \right)}^{-1.166}$ Extrapolated $k_{Re10}=\;2.041*10^{-9}*{\left(1-\varphi \right)}^{-1.002}$	R^2^.9700 R^2^.9477

## Discussion

**Figure 12.**
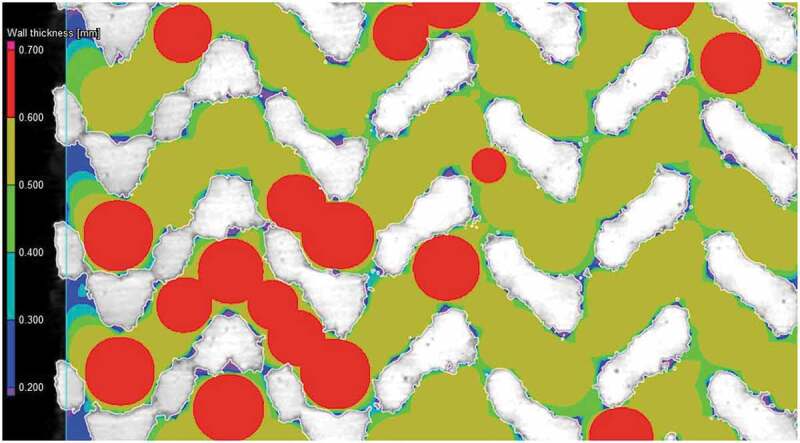
Surface roughness and sintered particles’ impact on pore diameter measurement of the diamond lattice. The scanned structure in white and pore evaluation is color-coded

Vertical shrinkage identified during the geometrical analysis of all the specimens of this study is explained by the use of a rubber blade in the powder recoating assembly of a Trumpf LPBF system. Contrary to less compliant metallic blades, rubber blades allow positive vertical deformations caused by thermal stresses, and result in lesser compressed, and therefore, more vertically shrunk structures. In the manufactured structures, the overall porosity is lower than designed, which can be partly attributed to the powder particles sintered to the surface. In order to get rid of the sintered particles and come closer to the desired porosity, a more thorough cleaning or etching may be warranted. This effect is also responsible for the smaller than designed pore diameters as seen in Figure 12. Another concern regarding weakly-bonded particles is the risk of their loosening after implantation which could pose health problems from ion release in other parts of the body. High concentrations of powder particles (>1x10^5^ particles/mL) are known to affect cell viability indicating the need to minimise the quantity of surface-sintered particles [71].

Among the three porosity measurement techniques used in this study, each has its own set of drawbacks. The porosity value resulting from CT-scans is highly dependent on the threshold value used. The porosity values measured using ASTM F2450 are affected by the diameter and height measurements, which, in their turn may be overestimated due to the as-built surface roughness. Lastly, the porosity obtained using Archimedes’ technique (ASTM B963) depends on the quality of the oil impregnation and weighting procedures (all voids must be filled with oil and this oil must be kept inside the specimen during all the manipulations to improve precision of these measurements).

The deviations in terms of the strut/sheet thickness and pore diameter between the manufactured and designed specimens can be further explained by the fact that the preliminarily determined 70 µm laser compensation is only viable for the X-Y manufacturing plane and not in the Z build direction, where the melt pool depth and not the laser path, determines the process accuracy. To correct the build direction accuracy would require a tuning of the printing parameters such as the laser power and the scanning speed. Some studies have noted this effect and developed an optimization procedure to compensate for such a discrepancy [52]. The gyroid lattice manufactured with the highest target porosity of 80% presents the largest deviations from the designed structure which can affect the testing results, notably the fluidic permeability. Nonetheless, a porosity range of ~60–75% and a pore diameter range of 550–660 μm of the manufactured structures fall within the recommended ranges for porous implants - 30–70% porosity and 100–1000 μm pore diameter range. It can be hypothesized that spinal cages integrating such porous structures would favor osseointegration, and therefore, enhance the implant fixation quality.

**Figure 13.**
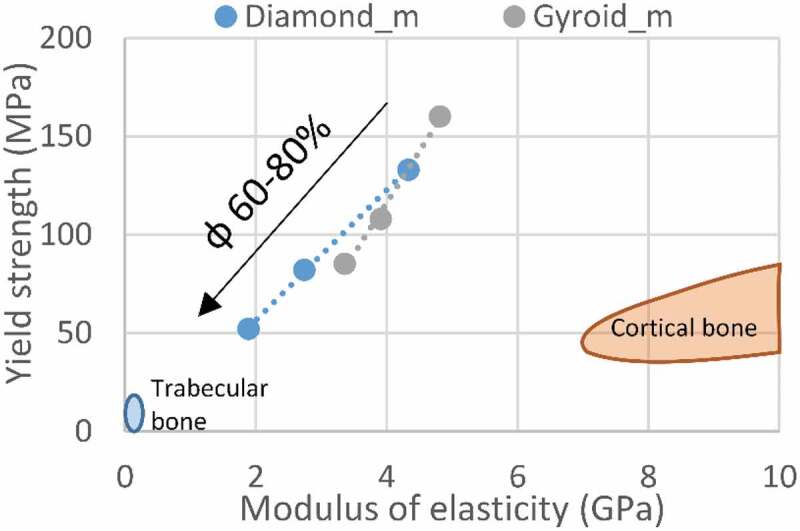
Yield strength vs modulus of elasticity comparison of the manufactured structures and bone tissues

The mechanical properties of the manufactured structures also correspond to the defined set of functional requirements (Table 4). The modulus of elasticity, ranging from 1.9 to 4.8 GPa, is situated between that of cortical bone (7–30 GPa) and that of trabecular bone (0.043–0.165 GPa). Next, the yield stress of the lattice structures (52–160 MPa) is in the same range as that of cortical bone (42–176 MPa), being from 5- to 15– times higher than that of trabecular bone (0.2–10.5 MPa) (Figure 13).

**Table 4.** Mechanical properties of biological tissues, commonly used spinal cage materials and manufactured lattice structures

			Sy/failure stress* [MPa]	Young’s Modulus E [GPa]	*Sy*/*E* [x 10^−3^]	*S*/*V* [mm^−1^]	Permeability [x 10^−9^ m^2^]
Cortical bone			42–176	7–30	5.9–6	2–4	0.5–44.5
Trabecular bone			0.2–10.5	0.043–0.165	5–60	1–6	
Vertebrae			3–6*	0.374	8–16	2–3	
PEEK [72]			97.5	3.9	25	-	-
Ti-6Al-4V [53]			1120	114	9.8	-	-
Ti64 Diamond lattice	φ [%]	61.2	133	4.3	30.7	3.43	5.71–41.7
		69.6	82	2.7	30.0	3.50	8.26–53.9
		76.3	52	1.9	27.6	3.52	9.84–57.2
Ti64 Gyroid lattice	φ [%]	59.2	160	4.8	33.3	3.04	5.15–32.0
		69.0	108	3.9	27.6	3.35	7.28–46.9
		72.3	85	3.4	25.3	3.66	7.55–50.1

At higher levels of porosity, the yield strength of the diamond structures is 52 MPa and that of the gyroid structures, 85 MPa, meaning that a porous spinal cage can cover only ~12% of the vertebral face contact area and still be as resistant as the vertebrae itself. This can allow the use of smaller spinal devices, requiring less invasive surgery and easier rehabilitation. However, such smaller devices would result in higher local stresses in surrounding vertebrae, increasing the risk of implant subsidence and bone failure. Therefore, the porous implant must be optimized to facilitate surgery, while reducing such risks of post-surgery complications.

**Figure 14.**
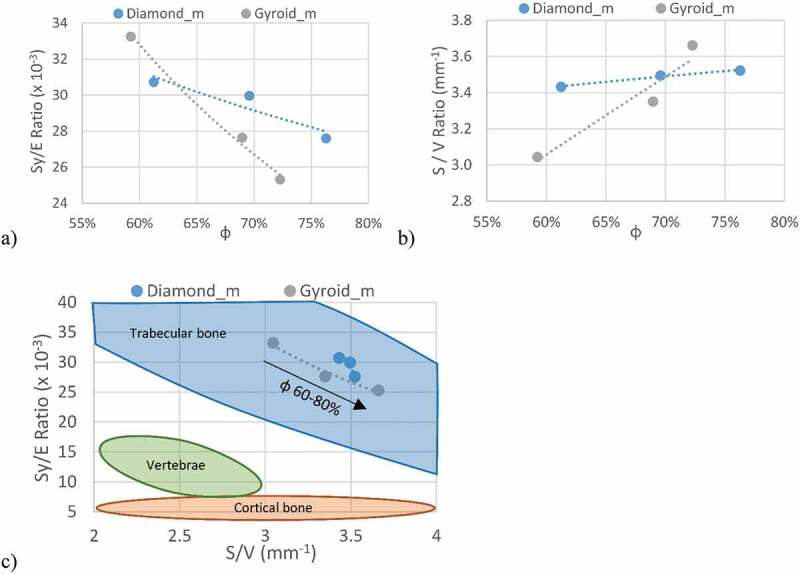
(a) Strength-to-stiffness, (b) surface-to-volume ratios of the manufactured structures and (c) their comparison with bone tissues in the Sy/E – S/V space

Strength-to–stiffness ratios of 25–33 × 10^−3^ are achieved with the lattice structures of this study, which are much higher than those of bulk metals, having *Sy*/*E* ratios of ~10 × 10^−3^, and are in the same range as PEEK at ~25 × 10^−3^ (Table 4). Compared to fully dense Ti-6Al–4V, this represents a 24–60–fold decrease in the modulus of elasticity, while only reducing the yield stress by a factor of 7–21, indicating that the porous structures greatly outperform their bulk equivalents currently used in spinal devices. The strength-to-stiffness ratios of the diamond lattices are slightly lower than those of the gyroid lattices at lower porosities, while at higher porosities, the situation is reversed (Figure 14(a)). This effect originates from the stiffness of gyroid structures which is less influenced by porosity variations as compared to the diamond structures (Figure 9). Though it may seem counterintuitive, the *Sy/E* ratio of the gyroid lattice is more porosity–sensitive than that of its diamond equivalent. For example, when *φ* increases from 60 to 80%, the *Sy/E* ratio of the former decreases by ~24%, while the latter, only by ~10%. Since the *Sy/E* criterion should be maximized, the gyroid lattice is more suited for porosities lower than ~63%, and the diamond lattice, for porosities above this threshold. When compared to the *Sy/E* of bone tissues (Figure 14(c)), the lattice structures exhibit higher ratios than cortical bone (~6 × 10^−3^) and vertebrae (8–16 × 10^−3^), but are situated in the same range as trabecular bone (5–60 × 10^−3^).

The *S/V* ratios of all the manufactured structures are higher than the designed values (Table 2 and Figure 14(b)), which is mainly due to sintering of powder particles to the part surface. At lower porosities, the gyroid lattices exhibit smaller *S*/*V* values than the diamond lattices, but overtake them at ~70% (Figure 14(b)). Since a higher surface-to-volume ratio is better for the application, for porosities under 70%, diamond structures appear to be more advantageous, while above 70%, gyroid structures prevail. As with the *Sy/E* criterion, the *S/V* ratio of the diamond structures is less porosity–dependent (~3% increase when *φ* increases from 60 to 80%) than that of the gyroid structures (~15% increase). Finally, all the studied structures have *S/V* ratios in the range of 3–4 mm^−1^, which corresponds to the mean *S/V* value of trabecular bones, and is significantly higher than that of vertebrae (Figure 14(c)).

The permeability extrapolated to the Darcy regime range (Table 4) indicates that the diamond structures are more permeable across the range of porosities of this study, which is in agreement with previous research showing that strut-based lattices are more permeable than their sheet–based equivalents [42]. Nonetheless, all the obtained permeability values range from 5.15 to 57.2 × 10^−9^ m^2^, thus covering the reported vertebrae permeability range of 0.49 to 44.5 × 10^−9^ m^2^.

The difference in the *Sy/E* and *S/V* ratios between the two structures is fairly small as compared to the ranges covered by bone tissues. In addition, the two criteria indicate some ambiguity and do not allow selecting a better candidate between the two analyzed structures. While the permeability results indicate that the diamond structures are marginally better suited for spinal cages applications than the gyroid structures, the latter are stronger and contain a lesser amount of stress risers as demonstrated by their respective failure modes. In fact, given the small difference of *Sy/E* values between the two structures, the gyroid structures, which manifest higher mechanical resistance, appear to be more advantageous. In addition, according to the literature [43], the less pronounced stress riser effect could bring an even more significant advantage of gyroid structures in terms of their fatigue resistance.

While a comparison between the present results and other studies could be beneficial, there is a large disparity between the current work and the literature in terms of lattice design, porosity level and pore size. This discrepancy results in a limited overlap between studies, rendering direct comparisons almost impossible. Notwithstanding that, the mechanical response of the ~60% porous gyroid structure studied in this work (*E*= 4.8 GPa and *Sy *= 160 MPa) is very close to that of the ~60% porous gyroid structures in Bobbert et al. [52]: *E*= 4–6 GPa and *Sy *= 150–200 MPa. Furthermore, the fluidic permeability of gyroid structures of this study (5–50 × 10^−9^ m^2^) is of the same order of magnitude as its equivalents in Bobbert et al. [52] (1–3 × 10^−9^ m^2^), Ma et al. [51] (0.25–5 × 10^−9^ m^2^) and Castro et al. [69] (25–120 × 10^−9^ m^2^).

This study is aimed at filling the gap in terms of a concurrent assessment of the geometric, mechanical and fluid permeability properties of the diamond- and gyroid–based lattice structures with identical porosities and pore sizes, and their conformity with the functional requirements of spinal cages. Among the limitations of this study are a relatively limited range of analyzed porosities (60–80%) and the application of just the compression mechanical testing mode. In the continuation, in addition to the mandatory fatigue testing, the mechanical properties must be evaluated not only for compression, but also for bending and torsion, since these testing modes approximate the real loading conditions in the spine.

## Conclusion

This experimental study covered the selection and comparison of two competing lattice structures for use in intervertebral cages. Their geometric, mechanical and fluid permeability properties were analyzed experimentally and compared to spinal implants functional requirements. Results reveal that the above-mentioned attributes of the diamond and gyroid lattices fall within the established requirements, providing potential solutions for reducing the complication rates of spinal devices by offering a better fixation through osseointegration. The diamond and gyroid lattice structures provide very similar results under this study’s testing conditions. The major differences are in the failure mode, which consists of sudden buckling of certain struts in the diamond lattice, while the gyroid lattice exhibits a more progressive failure via the collapse of lattice walls. This would seem to indicate, as previously stipulated, that stresses are distributed more uniformly in the gyroid lattices than in their diamond equivalents, which promise greater fatigue resistance. The stiffness of both structures (1.9–4.8 GPa) is much lower than that of the dense material used for their manufacture (~100 GPa), while their mechanical resistance (52–160 MPa) is greater than that of vertebrae (3–6 MPa), thus decreasing the risks of bone and implant failure. The small differences between the *Sy/E* (25–33 × 10^−3^) and *S/V* (3–4 mm^−1^) ratios of both structures make it difficult to select a better–suited candidate for the application. The fluid permeability (5–57 × 10^−9^ m^2^) and *S/V* ratios of both lattice structures are close to those of vertebrae, promising an adequate osseointegration. Further work should focus on the fatigue resistance of the gyroid and diamond lattice under different loading modes, including compression, tension and torsion.

## Data Availability

The raw/processed data required to reproduce these findings cannot be shared at this time as the data also forms part of an ongoing study.
